# Effect of Planting Density and K_2_O:N Ratio on the Yield, External Quality, and Traders' Perceived Shelf Life of Pineapple Fruits in Benin

**DOI:** 10.3389/fpls.2021.627808

**Published:** 2021-06-16

**Authors:** Ulrich Djido, Nicodeme V. Fassinou Hotegni, Willemien J. M. Lommen, Joseph D. Hounhouigan, Enoch G. Achigan-Dako, Paul C. Struik

**Affiliations:** ^1^Laboratory of Genetics, Biotechnology and Seed Science, Faculty of Agronomic Sciences, University of Abomey-Calavi, Cotonou, Benin; ^2^Centre for Crop Systems Analysis, Wageningen University and Research, Wageningen, Netherlands; ^3^Laboratory of Food Sciences, Faculty of Agronomic Sciences, University of Abomey-Calavi, Cotonou, Benin

**Keywords:** potassium fertilization, K_2_O:N ratio, quality attributes, intercropped pineapple, shelf life, planting density, Benin, *Ananas comosus* var. *comosus*

## Abstract

Quality, shelf life, and yield of a pineapple fruit are the important attributes for the producers and customers in the pineapple value chain of Benin, whereas poor quality, short shelf life, and low yield are the main constraints. We quantified the effects of planting density and K_2_O:N fertilizer ratio on the pineapple yield, external quality, and perceived shelf life in four on-farm experiments with cv. Sugarloaf in Benin; two experiments were installed in the long rainy season and two in the short rainy season. A split-plot design was used with the planting density as the main factor at three levels: 54,000, 66,600, and 74,000 plants.ha^−1^. The K_2_O:N ratio was a subfactor with three levels: K_2_O:N = 0.35 (farmers' practice), K_2_O:N = 1, and K_2_O:N = 2. The results showed that both factors had no effect on the crop development variables (such as the number of functional leaves and D-leaf length) at the moment of flowering induction. The planting density had no effect on the total weight per fruit, infructescence weight, total fruit length, infructescence length, crown length, or the fruit shelf life as perceived by traders. The yield increased from 54.9–69.1 up to 90.1 t.ha^−1^ with an increase in the planting density. The yield increase was not at the expense of the fruit weight. Increased K_2_O:N ratio led to a higher fruit weight whereas the fruit length was not affected. The shelf life of fruits produced at a K_2_O:N ratio of 1 and as perceived by traders was 6 days longer than that of fruits produced at a ratio of 0.35 (farmers' practice). Based on these results, we suggest the fresh pineapple farmers in Benin to use a combination of 66,600 plants.ha^−1^ with a K-fertilization scheme based on a K_2_O:N ratio of 1 to meet the expectation of both producers and customers in terms of fruit yield and fruit quality.

## Introduction

Pineapple *(Ananas comosus* [L.] Merrill) is a fruit predominantly produced in (sub)tropical countries. The fruit contributes to more than 20% of the tropical fruit production worldwide with 25.5 million tons (FAO, 2016); it contains vitamins, such as vitamin A, B1, B6, and C, as well as other nutrients, such as copper, manganese, and fibers (Morton, 1987; Mateljan, 2007; Pérez et al., 2011). There are eight countries in West Africa producing pineapples, with Benin being the third largest fresh pineapple producer (next to Ghana and Nigeria) in 2018 with 372,507 tons (FAO, 2018). According to Houessou (2017), pineapple production contributes to 1.2% of the Gross Domestic Product (GDP) and 4.3% of the agricultural GDP in Benin. Fassinou Hotegni et al. (2014) reported that pineapple is a main crop in the southern part of Benin, especially in the Atlantic Department where it is cultivated by about 70% of the farmers. The Atlantic Department realizes about 95% of the national pineapple production in Benin (Karim et al., 2018). Two pineapple cultivars are widely grown in Benin: “Smooth Cayenne” and “Sugarloaf,” with “Sugarloaf” being the more widely cultivated of the cultivars (Fassinou Hotegni et al., 2014). In 2016, the Beninese government, through its Governmental Actions Plan (PAG), decided to include the pineapple fruit as one of the most important crops to be promoted with a target of 600,000 tons by 2021. Meanwhile, Mba (2019) reported poor quality of the fresh pineapple fruits, which leads to a low proportion of marketable fruits, as a major bottleneck in the fresh pineapple supply chain in Benin; traders mentioned a short shelf life of the delivered fresh pineapple (<3 days). Therefore, to reach the target set by the government, an increase in crop yield along with improved fruit quality (including shelf life) through improved and sustainable agronomic practices is needed.

Many studies have been carried out to increase the pineapple fruit yield (Dalldorf, 1992; Mohammed Selamat, 1995; Agbangba, 2008; Hung et al., 2011; Agbangba et al., 2015; Sossa et al., 2017). Actual crop yield is determined by many factors including planting material (genetic quality and planting material health and vigor), environmental conditions, agronomic practices (planting density and fertilizer management), pests and diseases, and their interactions (Momoh and Zhou, 2001; Tollenaar and Lee, 2002). In the current pineapple production systems in Benin, the planting density varies greatly between 40,000 and 90,000 plants.ha^−1^ and different plant arrangements are used at planting (quincunxes, beds of two alternating rows, and single rows) (Fassinou Hotegni et al., 2012); also the fertilizer management was found not to be well-balanced with a high variation in farmers' practices (K_2_O:N ratio between 0.30 and 3.8) (Agbangba et al., 2011).

In the fruit crop production, the planting density affects greatly the crop yield and fruit quality and varying effects have been reported. In the pineapple cultivation, Thomas and Dodson (1968) found that increasing the planting density of the pineapple cv. Cayenne from 42,400 to 102,500 plants.ha^−1^ increases the yield per hectare but reduces the fruit size and sucker production per plant. These results have been confirmed later by Norman (1977) and Luning et al. (2002), who found that increasing the planting density also reduced the number of leaves. Dass et al. (1978) studying the response of cv. Kew (equivalent to cv. Cayenne in Hawaii) rather found that increasing the planting density did not affect the number of leaves. With the same cultivar (cv. Kew), Hung et al. (2011) found that increasing the planting density up to 78,000 plants.ha^−1^ increased the plant height and decreased the width of the D-leaf (the longest leaf in a pineapple plant according to Malézieux et al., 2003) as well as the percentage of plants responding to the flowering induction agent after artificial induction of flowering. According to the same authors, the optimum density in the pineapple cultivation is 66,000 plants.ha^−1^. For cv. Sugarloaf, Norman (1978) showed that increasing the planting density from 17,000 to 57,000 plants.ha^−1^ increased the total fruit yield but decreased the fruit weight and length, peduncle thickness, and slip number per plant. The same author also found that increasing the planting density in cv. Sugarloaf did not affect internal quality attributes, such as total soluble solids (TSS), pH, and percentage of titratable acidity. Maia et al. (2009), working on cv. Perola, a cultivar close to cv. Sugarloaf, found that increasing the planting density from 41,666 to 55,555 plants.ha^−1^ had no significant effects on the fruit diameter, length, and firmness. Mohammed Selamat (1995) working with cv. Gandul found that increasing the planting density from 43,056 to 61,508 plants.ha^−1^ reduced the number of leaves; but no effect was observed on the plant height and D-leaf length, average fruit weight, and fruit length. In the context of the pineapple production in many countries including Benin where the pineapple is grown mainly together with an intercrop like maize (*Zea mays* L.), cassava (*Manihot esculenta* Crantz), chili pepper (*Capsicum annuum* L.), or tomato (*Solanum lycopersicum* L.) (Fassinou Hotegni et al., 2012), and where no studies on the pineapple planting density in relation to the crop yield and fruit quality including the shelf life have been reported so far, there is a need to define the optimum planting density considering the crop yield and fruit quality, including the shelf life.

Regarding the fertilizer management in pineapple cultivation, macronutrients, such as nitrogen (N), phosphorus (P), and potassium (K) affect not only the vegetative phase of the pineapple but also the reproductive phase. Malézieux and Bartholomew (2003) reported that the leaf number and average leaf size are reduced when N is deficient, leading to a reduction in the fruit weight. According to Malézieux and Bartholomew (2003), the amount of N needed for the pineapple cultivation ranges from 250 to 700 kg.ha^−1^ (4–10 g N per plant) depending on the planting density, the soil condition, and the expected fruit weight. Phosphorus is important for the root system and the growth of all parts of the plant because phosphorus is involved in root initiation (Malézieux and Bartholomew, 2003). Malézieux and Bartholomew (2003) also reported that the phosphorus requirement for the pineapple crop is low and the plant can extract P from the soil with low P content. Ma et al. (2013) found that P has a little effect on the pineapple fruit quality. Potassium is reported as the most important macronutrient, which determines mainly the fresh pineapple quality (Razzaque and Hanafi, 2001) and fruit firmness, a proxy of the fruit shelf life (Quaggio et al., 2009). Spironello et al. (2004) found that K has a positive effect on the pineapple yield and fruit size. Teixeira et al. (2011) working with the pineapple cv. Cayenne and comparing two sources of K reported that the fruit yield increased with an increase in the K fertilization (350–700 kg.ha^−1^ of K_2_O). In high rates of K applications, the fertilization with K sourced from K_2_SO_4_ showed better results than the fertilization with K from KCl. Quaggio et al. (2009) found that the K application regardless of the source improved the pineapple fruit shelf life. Malézieux and Bartholomew (2003) found that, in the pineapple cultivation, the potassium requirement ranges from 200 to 1,000 kg.ha^−1^ (~8–20 g K per plant). Considering the pineapple farmers' practices in Benin (Fassinou Hotegni et al., 2012) and in most pineapple-producing countries, N, P, and K are applied together; hence, some combined effects between these macronutrients on the quality of the pineapple have been reported mainly through the K_2_O:N ratio. For instance, Vllela-Morales et al. (1977) working on cv. Pernambuco found that the increased levels of N combined with the increased levels of K resulted in an increase in the fruit weight, plant height, and number of slips and suckers, but the increased levels of P did not affect these characteristics. Osei-Wusu (1995) and Owusu-Bennoah et al. (1995) working with cv. Cayenne in Ghana found that a K_2_O:N ratio of 2.5 at a low N level of 224 kg.ha^−1^ was adequate for the pineapple fruit production. According to Malézieux and Bartholomew (2003) and Hung et al. (2011), the K_2_O:N ratio should be around 2 for quality pineapple production.

Since the pineapple farmers apply in the field not only a certain density but also a certain fertilization scheme based on the K_2_O:N ratio, the single effect of a given practice (planting density or fertilization scheme) may be affected by the level of the other practices. So far, no scientific paper has reported the effect of both the planting density and fertilization scheme on the pineapple yield and quality (including the shelf life). Therefore, the objective of the present paper was to quantify the effect and the interaction between the planting density and a fertilizer management scheme based on the K_2_O:N ratio on the pineapple fruit yield, quality attributes (mainly the external pineapple quality), and shelf life, under farmers' conditions. The present paper is important since it will provide a clear-cut answer on how the planting density and K_2_O:N fertilizer ratio affect the fresh pineapple yield, external fruit quality, and shelf life.

## Materials and Methods

### Experimental Sites

**Figure 1:**
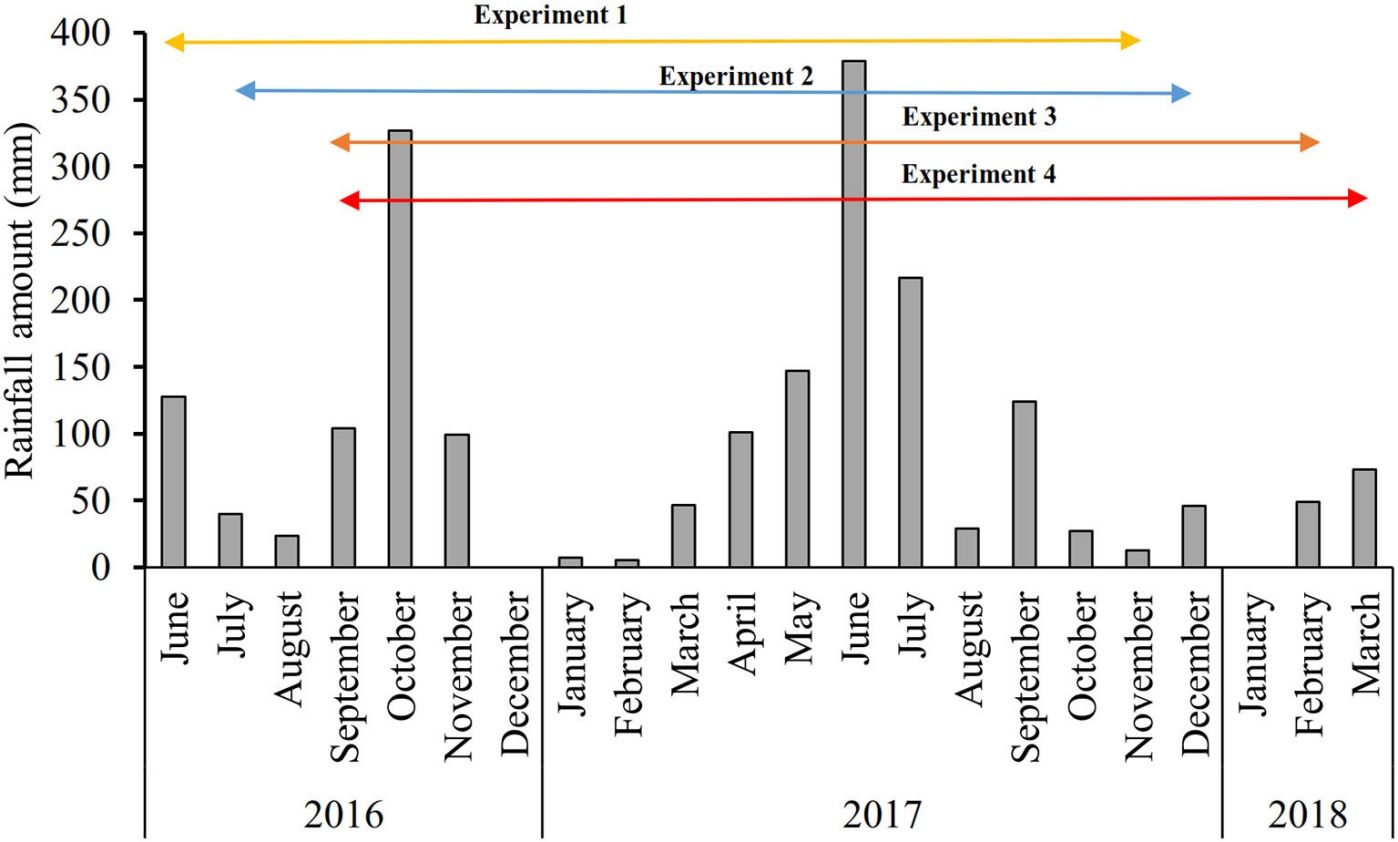
Variation in monthly rainfall during the experimentation period (June 2016 to March 2018) (Data collected from the International Institute of Tropical Agriculture IITA-Benin Station).

**Figure 2:**
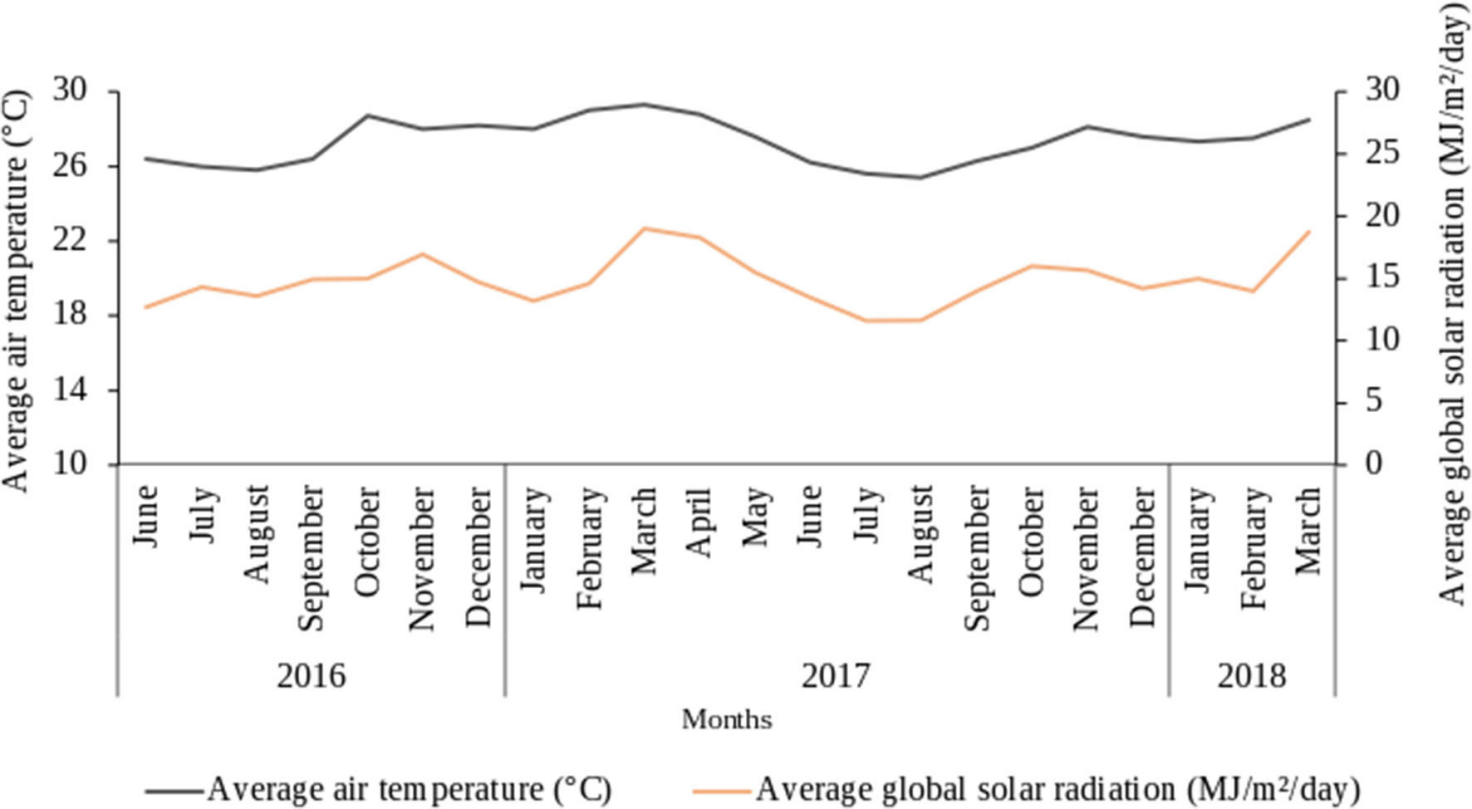
Variation of mean air temperature and solar radiation during the experimentation period (June 2016 to March 2018) (Data collected from the International Institute of Tropical Agriculture IITA-Benin Station).

Four on-farm experiments were conducted in the Atlantic department (municipalities of Abomey Calavi and Zè) in the south of Benin with cv. Sugarloaf. The municipalities of Abomey-Calavi and Zè were selected because of their proximity to the local and regional pineapple markets as well as a high number of fresh pineapple producers in these municipalities. Two experiments were set up during the long rainy season (in June 2016 and July 2016) and two during the short rainy season (September 2016) since Fassinou Hotegni et al. (2012) reported that most pineapple farmers preferred to plant pineapple in the rainy seasons. Before the experiments were set up, composite soil samples were collected at each experimental site for soil analysis. Information on the fields and cultural practices is provided in Table 1. It is important to point out that maize (*Z. mays* L.) was sown within the bands of alternating rows of pineapple just after the pineapple planting following the farmers' practices (89% of the pineapple producers intercropped pineapple with maize, as reported by Fassinou Hotegni et al., 2012). Information on the fertilizer application time and doses is provided in Table 2. Information on the monthly climatic data including rainfall amount, mean air temperature, and average solar radiation during the experimentation period are provided in Figures 1, 2. The mean monthly rainfall was 96 and 100 mm respectively for experiments 1 and 2 (long rainy season planting) and 94 mm for experiments 3 and 4 (short rainy season planting). Temperature sum (Tsum) over the crop phenological stages per experiment was also computed (Table 3) by using the following formula:

(1) $$\begin{array}{r} Tsum=\overset{i=d}{\underset{i=1}{\sum }}\left(T_{i}-T_{b}\right) \end{array}$$

*T*_i_ = Average daily temperature during the experimentation period (*T*_i_ was always higher than *T*_b_ throughout both growing seasons).

**Table 1:** Field information, cultural practices including the levels of planting density in the four experiments.

**Information on the fields**	**Experiment 1/Long rainy season**	**Experiment 2/Long rainy season**	**Experiment 3/Short rainy season**	**Experiment 4/Short rainy season**
Municipality (Village)	Zè (Djissoukpa)	Abomey-Calavi (Zinvé- Kpé)	Abomey-Calavi (Wawatta)	Zè (Glodjissoukpa)
Soil type (U.S. equivalent)	Ferralic soil (Ultisols)	Ferralic soil (Ultisols)	Ferralic soil (Ultisols)	Ferralic soil (Ultisols)
Climate	Subequatorial	Subequatorial	Subequatorial	Subequatorial
Planting time	17th June 2016 (Long rainy season)	17th July 2016 (Long rainy season)	09th September 2016 (Short rainy season)	14th September 2016 (Short rainy season)
Types of planting material used	Slips (325–525 g)	Slips (325–525 g)	Slips (325–525 g)	Slips (325–525 g)
Maize sowing time	30th August 2016 (Short rainy season)	1st September 2016 (Short rainy season)	12th September 2016 (Short rainy season)	No maize^a^
Plant arrangement at planting	Flat beds of two alternating rows	Flat beds of two alternating rows	Flat beds of two alternating rows	Flat beds of two alternating rows
Planting spacing: BP^b^ × BR^c^/BDR^d^ (cm)	According to treatment: 35 × 45/60; 30 × 40/60; 30 × 40/50	According to treatment: 35 × 45/60; 30 × 40/60; 30 × 40/50	According to treatment: 35 × 45/60; 30 × 40/60; 30 × 40/50	According to treatment: 35 × 45/60; 30 × 40/60; 30 × 40/50
Planting density (plants/m^2^)	5.44; 6.66; 7.40	5.44; 6.66; 7.40	5.44; 6.66; 7.40	5.44; 6.66; 7.40
Maize harvest time	31th December 2016 (Short rainy season)	27th December 2016 (Short rainy season)	05th January 2017 (Short rainy season)	NA^f^
Artificial flowering induction time	12 MAP^e^ (21 June 2017)	12 MAP (17 July 2017)	12 MAP (9 September 2017)	12 MAP (25 September 2017)
Weed control	Hand weeding	Hand weeding	Hand weeding	Hand weeding
Harvest time	17 MAP + 11 days (28–29 November 2017)	17 MAP – 4 days (13–14 December 2017)	17 MAP (9–10 February 2018)	18 MAP – 6 days (6 March 2018)

a *Maize plants were destroyed several times after emergence by domestic animals until the rain stopped*.

b *BP, spacing between plant within a row*.

c *BR, spacing between rows*.

d *BDR, spacing between double rows*.

e *MAP, months after planting*.

f *NA, not applicable*.

**Table 2:** Field information on fertilizer application scheme for cv. sugarloaf in the four experiments.

**Subplot factor: fertilizer**	**Types of fertilizer applied and dose (D) per plant**	**N per plant (g)**	**K_**2**_O per plant (g)**	**Experiment 1/Long rainy season**	**Experiment 2/Long rainy season**	**Experiment 3/Short rainy season**	**Experiment 4/Short rainy season**
K_2_O:N = 0.35	First Urea (46%N) + NPK (15-15-15); D = 7.36 g + 3.66 g	3.94	0.55	3 MAP^a^ (17 September 2016)	3 MAP (17 October 2016)	6 MAP (9 March 2016)	6 MAP (14 March 2016)
	Second Urea (46%N) + NPK (15-15-15); D = 3.67 g + 7.33 g	2.79	1.10	9 MAP (17 March 2017)	9 MAP (17 April 2017)	6 MAP (9 March 2016)	6 MAP (14 March 2016)
	NPK (15-15-15) 7.33 g	1.10	1.10	9 MAP (17 March 2017)	9 MAP (17 April 2017)	9 MAP (9 June 2017)	9 MAP (14 June 2017)
K_2_O:N = 1: previous treatment + one application of K_2_SO_4_	First Urea (46%N) + NPK (15-15-15); D = 7.36 g + 3.66 g	3.94	0.55	3 MAP (17 September 2016)	3 MAP (17 October 2016)	6 MAP (9 March 2016)	6 MAP (14 March 2016)
	Second Urea (46%N) + NPK (15-15-15); D = 3.67 g + 7.33 g	2.79	1.10	9 MAP (17 March 2017)	9 MAP (17 April 2017)	6 MAP (9 March 2016)	6 MAP (14 March 2016)
	NPK (15-15-15); D = 7.33 g	1.10	1.10	9 MAP (17 March 2017)	9 MAP (17 April 2017)	9 MAP (9 June 2017)	9 MAP (14 June 2017)
	**First K**_**2**_**SO**_**4**_ **(50% K**_**2**_**O); D=10.16 g**	**Not applied**	**5.08**	**9 MAP (17 March 2017)**	**9 MAP (17 April 2017)**	**9 MAP (9 June 2017)**	**9 MAP (14 June 2017)**
K_2_O:N = 2: previous treatment + two applications of K_2_SO_4_	First Urea (46%N) + NPK (15-15-15); D = 7.36 g + 3.66 g	3.94	0.55	3 MAP (17 September 2016)	3 MAP (17 October 2016)	6 MAP (9 March 2016)	6 MAP (14 March 2016)
after the flowering induction	Second Urea (46%N) + NPK (15-15-15); D = 3.67 g + 7.33 g	2.79	1.10	9 MAP (17 March 2017)	9 MAP (17 April 2017)	6 MAP (9 March 2016)	6 MAP (14 March 2016)
	NPK (15-15-15); D = 7.33 g	1.10	1.10	9 MAP (17 March 2017)	9 MAP (17 April 2017)	9 MAP (9 June 2017)	9 MAP (14 June 2017)
	First K_2_SO_4_ (50% K_2_O); D = 10.16 g	Not applied	5.08	9 MAP (17 March 2017)	9 MAP (17 April 2017)	9 MAP (9 June 2017)	9 MAP (14 June 2017)
	**Second K**_**2**_**SO**_**4**_ **(50% K**_**2**_**O); D** **=** **7.83 g**	**Not applied**	**3.91**	**1 WAF^b^ (24 June 2017)**	**1 WAF (24 July 2017)**	**1 WAF (16 September 2017)**	**1 WAF (21 September 2017)**
	**Third K**_**2**_**SO**_**4**_ **(50% K**_**2**_**O); D** **=** **7.83 g**	**Not applied**	**3.91**	**3 MAF^c^ (24 September 2017)**	**3 MAF (24 October 2017)**	**3 MAF (16 December 2017)**	**3 MAF (21 December 2017)**
Application form	Urea (46% N), NPK (15-15-15) and K_2_SO_4_ (50% K_2_O)	Solid at the base of the plants	Solid at the base of the plants	Solid at the base of the plants	Solid at the base of the plants	Solid at the base of the plants	Solid at the base of the plants

a *MAP, months after planting*.

b *WAF, weeks after the flowering induction*.

c *MAF, months after the flowering induction*.

*Texts in bold indicate additional applications compared to the previous treatment*.

**Table 3:** Thermal time (Tbase = 10°C) and duration of the total cycle, vegetative phase and generative (flowering and fructification) phase of pineapple in the different experiments.

**Experiments**	**Total T sum (degree days)**	**Total number of days**	**T sum during vegetative phase (degree days)**	**Number of days during vegetative phase**	**T sum during generative phase (degree days)**	**Number of days during generative phase**
Experiment_1	9116	531	6463.2	370	2652.6	161
Experiment_2	8897	517	6364.3	366	2532.4	151
Experiment_3	9047	520	6348.8	366	2698.4	154
Experiment_4	9567	539	6550.1	377	3017.2	162

*Temperature data collected from the International Institute of Tropical Agriculture (IITA)-Benin station*.

*T*_b_ = Base temperature for pineapple growth (10°C) according to Bartholomew and Criley (1983), Malézieux et al. (1994), and Py et al. (1987).

### Artificial Flowering Induction and Harvesting Time

All plants were artificially induced to flower by using carbide of calcium (CaC_2_) (which releases acetylene) at 12 months after planting (MAP) and following the farmers' practices; 50 ml of a solution containing 10 g.l^−1^ was applied at the center of the leaf rosette. Pineapple fruits were harvested at 150, 154, 154, and 154 days after the flowering induction in Experiments 1, 2, 3, and 4, respectively (Table 1). The harvesting time was determined by following the criteria for harvesting used by the fresh pineapple traders involved in the experiment. They visited the experiments when the fields were close to harvesting and their criteria were based on their appreciations of the skin color (eyes on the skin starting to turn yellow), eyes development on the fruit (smooth skin), and TSS (at least 12° Brix).

### Experimental Design

At each experimental site, a split-plot design with two factors was used. The main factor was the planting density with three levels following different plant arrangements at planting: (1) 35 × 45/60 cm (35 cm between the plants in a single row, 45 cm between the two single rows, and 60 cm between the double rows) corresponding to 5.44 plants.m^−2^ (54,400 plants.ha^−1^, farmers' practice), (2) 30 × 40/60 cm corresponding to 6.66 plants.m^−2^ (66,600 plants.ha^−1^), and (3) 30 × 40/50 cm corresponding to 7.40 plants.m^−2^ (74,000 plants.ha^−1^). The subplot factor was the amount of K provided per plant, which had three levels: applied at 2.75, 7.83, and 15.66 g K_2_O per plant (Table 2). Multiple amounts of K were used to obtain the ratios of K_2_O:N = 0.35 (farmers' practice) K_2_O:N = 1, and K_2_O:N = 2. The amounts of nitrogen (N) and phosphorus (P) were similar in all treatments and were 7.83 g N and 2.00 g P_2_O_5_ per plant over the crop cycle as applied by the farmers (Table 2). N and P were sourced from Urea (46% N) and NPK 15-15-15. The P level was very close to that found by Agbangba et al. (2015) to be optimum for cv. Cayenne. K was first sourced from the NPK (15-15-15) and later from the sulfate of potash (K_2_SO_4_, 50% K_2_O). Details on the timing and quantities of the fertilizers applied per treatment and plant are shown in Table 2. Four blocks and nine treatments per block (the combinations of planting densities and levels of K applications) were used. Each gross plot was composed of 10 rows of 42 plants each, giving 420 plants. Each net plot was composed of six lines of 10 plants each, giving 60 plants per net plot.

### Data Collection

Three types of data were collected: (1) data on the crop development status at the flowering induction, (2) data on the external fruit quality attributes and shelf life, and (3) data on the fresh pineapple yield.

Data on the crop development status included the number of functional (green) leaves, the length of the D-leaf (the longest leaf in a pineapple plant according to Malézieux et al., 2003), the (projected) D-leaf area, and the fresh weight of the D-leaf. These data were collected the day before the artificial flowering induction as a proxy of the crop development status at the moment of flowering induction. The number of functional leaves and the D-leaf length were recorded on all 60 plants of the net plot. The D-leaves of 6 randomly selected plants out of the 60 plants per net plot were removed and used to determine the D-leaf weight and the (projected) D-leaf area. The projected leaf area of the D-leaf was obtained by using a printer and the Mesurim-Pro image analysis software (Mouchet et al., 2011). The D-leaf was first divided into pieces and scanned by using the printer and next colored in green in the scanned file using Photoshop. The colored area was computed by using the software “Mesurim” to determine the leaf area of the D-leaf.

Data on external fruit quality were collected at the harvest time and included the infructescence weight, infructescence length, fruit weight, fruit length, crown weight, crown length, and the ratio of crown/infructescence length, on 25 plants per net plot after excluding the plants from which D-leaf was removed.

A shelf-life test was conducted by using the randomly selected four fruits per plot. The fruits were stored in the laboratory (Laboratory of Genetics, Biotechnology and Seed Science) between 24 and 26°C, and an ibutton (DS1922E, 15–40°C) was used and set to record the temperature every 10 min. The number of days until the fruits were no longer marketable was determined. This was done by traders with more than 20 years of experience in selling pineapple fruit who were asked to regularly visit the laboratory to inspect the fruits and to discard the fruits in case of no commercial value. This approach was used based on the co-creation approach (scientists and end users or stakeholders work together to generate knowledge/innovation). It was noticed that, over time, traders discarded the fruits showing less firmness with breaking down from the bottom and unpleasant odor.

The fresh pineapple yield was calculated from the total weight of fruits per net plot.

### Data Analysis

Data were analyzed by using the Genstat 19th edition. Before the data analysis, data were checked for the presence or absence of outliers. The outliers were first identified by using a scatter plot as suggested by Walfish (2006). The values of the outliers were checked against the original data set to ensure that these values were not the results of the effect of the different treatments before their removal (Sipes and Mendelsohn, 2001). Data from the plants on which the D-leaf was collected for the leaf area measurement by using the destructive measurement were also removed before the data analysis. To assess the effect of the planting density and K application on the (1) crop development status variables, (2) fresh fruit yield, and (3) external fruit quality (infructescence weight, infructescence length, fruit weight, fruit length, crown weight, crown length, and the ratio of crown/infructescence length), a two-way ANOVA for a split-plot design followed by a Least Significant Difference (LSD) test (α = 0.05) for the mean separation was used. Only the K fertilization levels based on K_2_O:N = 0.35 and K_2_O:N = 1 were used for analyzing the effects of the crop development status variables at the flowering induction because the third level K_2_O:N = 2 was applied after the flowering induction (Tables 1, 2). For the shelf-life test, the effects of the planting density and K_2_O:N ratio were depicted per cropping season (planting in long or short rainy seasons) through a two-way ANOVA using R.3.2.5 software (R CoreTeam, 2017). This was done since the fruits were collected in Experiments 1 and 3 only.

## Results

### Soil Analysis Before the Experimentation

The experimental sites were dominated by a red lateritic soil called “Terre de barre” classified as ferralsols (or Ultisol in US equivalent). Soils in all experimental sites were slightly acidic with a low soil organic matter content (<2%), the total nitrogen ranging from 0.3 to 0.1%, low cation exchange capacity (CEC <25 meq/100 g), and a low saturation rate (Table 4).

**Table 4:** Soil characteristics in the different experimental sites at different depths (0–20, 20–40, and 40–60 cm).

**Samples**	**pH**		**C_**org**_**	**N_**tot**_**	**P_**avail**_**	**CEC**	**K^**+**^**	**Na^**+**^**	**Ca^**2+**^**	**Mg^**2+**^**	**S**	**S/T**
	**Water**	**KCl**	**(%)**		**(ppm)**	**(meq/100 g of soil)**						**(%)**
**Depth 0–20 cm**												
Site 1_Exp. 1	4.65	4.32	1.41	0.10	10.32	22.50	2.66	2.64	0.54	7.38	13.22	58.76
Site 2_Exp. 2	5.45	4.62	1.46	0.04	16.55	26.25	1.35	1.14	0.54	4.66	7.69	29.30
Site 3_Exp. 3	6.56	5.89	1.36	0.06	23.26	15.63	0.31	0.11	0.73	0.85	2.00	12.80
Site 4_Exp. 4	5.73	5.50	1.05	0.06	28.21	18.75	0.31	0.30	0.54	0.85	2.00	10.66
**Depth 20–40 cm**												
Site 1_Exp. 1	4.88	4.30	1.01	0.06	14.96	18.12	1.84	1.53	0.41	3.57	7.35	40.56
Site 2_Exp. 2	4.74	3.99	0.86	0.03	12.62	11.25	0.49	0.16	0.41	1.28	2.34	20.80
Site 3_Exp. 3	5.08	4.52	0.95	0.04	18.14	13.13	0.17	0.32	0.27	1.06	1.82	13.86
Site 4_Exp. 4	5.11	4.79	0.72	0.05	20.87	13.13	0.13	0.07	0.41	1.17	1.78	13.56
**Depth 40–60 cm**												
Site 1_Exp. 1	4.70	4.26	0.62	0.02	44.57	15.00	0.31	1.69	0.68	5.09	7.77	51.80
Site 2_Exp. 2	5.07	4.90	0.74	0.01	13.39	15.00	1.21	1.40	0.82	5.31	8.74	58.27
Site 3_Exp. 3	4.79	4.25	0.66	0.03	18.53	16.25	0.17	0.50	0.14	0.74	1.55	9.54
Site 4_Exp. 4	4.84	4.54	0.54	0.03	19.30	11.25	0.08	0.20	0.14	0.63	1.05	9.33

*C_org_, organic carbon; N_tot_, total nitrogen; P_avail_, available phosphorus (Bray-1); CEC, cation exchange capacity; S, sum of bases; K^+^, potassium; Na^+^, sodium; Ca^2+^, calcium; Mg^2+^, magnesium; S/T, saturation rate*.

### Effects on Plant Characteristics at Flowering Induction

In all four experiments, both the planting density and K fertilization scheme had no significant effects on the number of functional leaves per plant and the D-leaf length at the moment of flowering induction (Table 5), nor were there any significant interactions between the two factors.

**Table 5:** Effects of planting density and K fertilization scheme before the flowering induction^1^ on the number of functional leaves per plant, the length, leaf area, and weight of D-leaf at the moment of flowering induction in the four experiments; data are presented as average ± SD.

**Source of variation**	**Exp. 1**	**Exp. 2**	**Exp. 3**	**Exp. 4**
**NUMBER OF FUNCTIONAL LEAVES PER PLANT**				
**Planting density (D)**				
Low (54,400 plants.ha^−1^)	24.30 ± 5.7	23.52 ± 5.5	18.55 ± 3.7	17.87 ± 4.2
Medium (66,600 plants.ha^−1^)	23.52 ± 6.7	22.58 ± 5.3	18.21 ± 4.4	17.99 ± 3.9
High (74,000 plants.ha^−1^)	24.09 ± 5.4	23.36 ± 5.9	17.80 ± 4.6	19.97 ± 4.5
*P*-value	ns	ns	ns	ns
**Fertilizer (F)**				
K_2_O:N = 0.35	23.18 ± 5.9	23.91 ± 5.4	18.54 ± 3.9	19.41 ± 4.4
K_2_O:N = 1	25.43 ± 5.8	23.22 ± 5.9	17.89 ± 4.1	18.33 ± 4.3
*P*-value	ns	ns	ns	ns
*P*-value interaction D × F	ns	ns	ns	ns
**D-LEAF LENGTH (cm)**				
**Planting density (D)**				
Low (54,400 plants.ha^−1^)	84.53 ± 13.2	94.84 ± 11.8	85.29 ± 8.2	88.03 ± 11.9
Medium (66,600 plants.ha^−1^)	81.91 ± 14.1	94.29 ± 12.7	86.90 ± 10.1	88.42 ± 9.9
High (74,000 plants.ha^−1^)	88.29 ± 14.0	95.14 ± 12.1	86.69 ± 8.9	91.49 ± 10.4
*P*-value	ns	ns	ns	ns
**Fertilizer (F)**				
K_2_O:N = 0.35	85.80 ± 14.1	93.99 ± 11.9	86.01 ± 9.3	89.35 ± 10.9
K2O:N = 1	84.84 ± 14.1	94.52 ± 12.3	86.62 ± 8.6	89.82 ± 9.9
*P*-value	ns	ns	ns	ns
*P*-value interaction D × F	ns	ns	ns	ns
**LEAF AREA OF D-LEAF (cm^2^)**				
**Planting density (D)**				
Low (54,400 plants.ha^−1^)	427.5 ± 83.0 b	453.6 ± 106.9	398.9 ± 70.2	424.7 ± 76.3
Medium (66,600 plants.ha^−1^)	436.1 ± 99.6 b	480.6 ± 106.1	409.2 ± 72.7	415.8 ± 87.4
High (74,000 plants.ha^−1^)	494.9 ± 102.9 a	477.2 ± 99.7	392.3 ± 75.4	402.9 ± 78.6
*P*-value	0.022^*^	ns	ns	ns
**Fertilizer (F)**				
K_2_O:N = 0.35	475.8 ± 90.2 a	459.4 ± 108.0	404.1 ± 66.7	403.5 ± 78.6
K_2_O:N = 1	434.4 ± 101.6 b	473.2 ± 101.9	400.7 ± 79.6	424.9 ± 85.2
*P*-value	0.050^*^	ns	ns	ns
*P*-value interaction D × F	ns	ns	ns	ns
**WEIGHT OF GREEN D-LEAF (g)**				
**Planting density (D)**				
Low (54,400 plants.ha^−1^)	68.6 ± 13.2 b	72.8 ± 11.8	60.2 ± 8.2	70.3 ± 11.9
Medium (66,600 plants.ha^−1^)	69.8 ± 14.1 b	78.0 ± 12.7	60.3 ± 10.1	68.0 ± 9.9
High (74,000 plants.ha^−1^)	80.7 ± 14.0 a	78.7 ± 12.1	57.6 ± 8.9	66.2 ± 10.4
*P*-value	0.048^*^	ns	ns	ns
**Fertilizer (F)**				
K_2_O:N = 0.35	77.1 ± 14.1	73.4 ± 11.9	59.9 ± 9.3	65.0 ± 10.9
K_2_O:N = 1	69.3 ± 14.1	75.7 ± 12.3	60.3 ± 8.6	70.7 ± 9.9
*P*-value	ns	ns	ns	ns
*P*-value interaction D × F	ns	ns	ns	ns

1 *K_2_O:N = 2 is not included because it became only different from K_2_O:N = 1 after the flowering induction. ns, not significant (p ≥ 0.05)*;

* *Significant at 0.01 ≤ p < 0.05. Means within a column followed by different letters are significantly different*.

The D-leaf area and D-leaf weight before the artificial flowering induction were not affected by the planting density and K fertilization scheme in three (Experiments 2–4) of the four experiments (Table 5). Only in Experiment 1, plants grown at high density (74,000 plants.ha^−1^) showed a higher leaf area and weight of the D-leaf than plants at medium and low planting densities. Regarding the K fertilization scheme in Experiment 1, the standard K fertilization scheme (K_2_O:N = 0.35) led to a higher D-leaf area than the K scheme application at a ratio of K_2_O:N = 1 (Table 5).

### Effects on Fruit Yield

The results showed significant main effects of the planting density and K_2_O:N ratio on the fruit yield per hectare (Table 6), and no significant interaction between these factors. The effect of the planting density on the pineapple fruit yield was consistent across experiments. In all experiments, yields per hectare increased with an increase in the planting density from 54,400 to 74,000 plants.ha^−1^. The increase in the yield from low planting density to high planting density varied between 25 and 33% (Table 6).

**Table 6:** Effects of the planting density and K_2_O:N ratio on the fresh fruit yield (Mg.ha^−1^) in the four experiments; data are presented as average ± SD.

**Source of variation**	**Exp. 1**	**Exp. 2**	**Exp. 3**	**Exp. 4**
**Planting density (D)**				
Low (54,400 plants.ha^−1^)	69.1 ± 7.1b	65.7 ± 6.1b	58.6 ± 3.6b	54.9 ± 5.1b
Medium (66,600 plants.ha^−1^)	81.4 ± 9.4a	76.3 ± 7.2a	72.9 ± 7.3a	65.0 ± 8.6a
High (74,000 plants.ha^−1^)	90.1 ± 6.7a	81.9 ± 5.4a	77.7 ± 6.2a	71.7 ± 10.5a
*P*-value	0.013^*^	0.008^**^	0.001^**^	0.015^*^
**Fertilizer (F)**				
K_2_O:N = 0.35	77.5 ± 10.8	72.7 ± 10.8b	66.2 ± 10.3b	58.6 ± 8.1b
K_2_O:N = 1	83.2 ± 9.5	73.4 ± 6.7b	72.4 ± 9.8a	64.2 ± 9.8a
K_2_O:N = 2	79.9 ± 13.9	77.8 ± 9.4a	70.6 ± 9.6a	68.9.±12.2a
*P*-value	ns	0.047^*^	0.038^*^	0.009^**^
*P*-value interaction D × F	ns	ns	ns	ns

*ns, not significant (p ≥ 0.05)*;

* *Significant at 0.01 ≤ p < 0.05*;

** *Significant at 0.001 ≤ p < 0.01. Means within a column followed by different letters are significantly different*.

An effect of the K_2_O:N ratio on the fruit yield was observed in three of the four experiments (Table 6). In Experiments 2–4, the yield of plants grown at a high ratio of K_2_O:N was 7–18% higher than the yield of plants grown at the standard ratio. In Experiment 1, no effect of K fertilization on the crop yield was observed.

### Effects on External Fruit Quality

#### Weight per Fruit, per Infructescence, and per Crown

In all experiments, the planting density did not significantly affect the total weight per fruit and the infructescence weight. The crown weight was only significantly affected in Experiment 2, where the plants grown at low planting density had a heavier crown than the plants grown at medium and high densities (Table 7).

**Table 7:** Effects of the planting density and K_2_O:N ratio on the weight per fruit, infructescence weight, crown weight, fruit length, infructescence length, crown length, and ratio crown: infructescence length in the four experiments; data are presented as average ± SD.

**Source of variation**	**Exp. 1**	**Exp. 2**	**Exp. 3**	**Exp. 4**
**FRUIT WEIGHT (kg)**				
**Planting density (D)**				
Low (54,400 plants.ha^−1^)	1.27 ± 0.2	1.21 ± 0.2	1.08 ± 0.2	1.01 ± 0.2
Medium (66,600 plants.ha^−1^)	1.22 ± 0.3	1.15 ± 0.2	1.10 ± 0.2	0.98 ± 0.2
High (74,000 plants.ha^−1^)	1.22 ± 0.2	1.11 ± 0.2	1.05 ± 0.2	0.97 ± 0.2
*P*-value	ns	ns	ns	ns
**Fertilizer (F)**				
K_2_O:N = 0.35	1.20 ± 0.2	1.12 ± 0.2 b	1.02 ± 0.2 b	0.91 ± 0.2 b
K_2_O:N = 1	1.28 ± 0.2	1.14 ± 0.2 b	1.11 ± 0.2 a	0.99 ± 0.2 ab
K_2_O:N = 2	1.23 ± 0.3	1.20 ± 0.2 a	1.09 ± 0.2 a	1.06 ± 0.2 a
*P*-value	ns	0.035^*^	0.036^*^	0.008^***^
*P*-value interaction D × F	ns	ns	ns	ns
**INFRUCTESCENCE WEIGHT (kg)**				
**Planting density (D)**				
Low (54,400 plants.ha^−1^)	1.14 ± 0.2	1.08 ± 0.2	0.96 ± 0.2	0.89 ± 0.2
Medium (66,600 plants.ha^−1^)	1.10 ± 0.3	1.03 ± 0.2	0.97 ± 0.2	0.86 ± 0.2
High (74,000 plants.ha^−1^)	1.09 ± 0.2	0.99 ± 0.2	0.95 ± 0.2	0.84 ± 0.2
*P*-value	ns	ns	ns	ns
**Fertilizer (F)**				
K_2_O:N = 0.35	1.08 ± 0.2	1.00 ± 0.2 b	0.92 ± 0.2	0.78 ± 0.2 b
K_2_O:N = 1	1.15 ± 0.2	1.02 ± 0.2 b	0.99 ± 0.2	0.88 ± 0.2 a
K_2_O:N = 2	1.10 ± 0.2	1.08 ± 0.2 a	0.97 ± 0.2	0.94 ± 0.2 a
*P*-value	ns	0.039^*^	ns	0.001^***^
*P*-value interaction D × F	ns	ns	ns	ns
**CROWN WEIGHT (g)**				
**Planting density (D)**				
Low (54,400 plants.ha^−1^)	0.13 ± 0.0	0.13 ± 0.0 a	0.12 ± 0.1	0.12 ± 0.0
Medium (66,600 plants.ha^−1^)	0.12 ± 0.0	0.11 ± 0.0 b	0.12 ± 0.1	0.11 ± 0.0
High (74,000 plants.ha^−1^)	0.13 ± 0.0	0.11 ± 0.0 b	0.10 ± 0.0	0.13 ± 0.0
*P*-value	ns	0.025^*^	ns	ns
**Fertilizer (F)**				
K_2_O:N = 0.35	0.12 ± 0.0	0.12 ± 0.0	0.11 ± 0.0	0.12 ± 0.0
K_2_O:N = 1	0.13 ± 0.0	0.12 ± 0.0	0.12 ± 0.1	0.12 ± 0.0
K_2_O:N = 2	0.13 ± 0.0	0.12 ± 0.0	0.12 ± 0.0	0.12 ± 0.0
*P*-value	ns	ns	ns	ns
*P*-value interaction D × F	ns	ns	ns	ns
**FRUIT LENGTH (cm)**				
**Planting density (D)**				
Low (54,400 plants.ha^−1^)	38.11 ± 3.7	34.24 ± 3.5	32.91 ± 3.5	29.38 ± 8.8
Medium (66,600 plants.ha^−1^)	37.73 ± 3.6	33.45 ± 3.7	32.59 ± 3.7	29.08 ± 3.3
High (74,000 plants.ha^−1^)	38.04 ± 3.4	32.90 ± 3.6	31.97 ± 3.5	29.10 ± 3.0
*P*-value	ns	ns	ns	ns
**Fertilizer (F)**				
K_2_O:N = 0.35	37.82 ± 3.7	33.31 ± 3.7	31.59 ± 3.8 b	28.78 ± 10.9
K_2_O:N = 1	38.19 ± 3.2	33.27 ± 3.7	33.23 ± 3.5 a	29.27 ± 9.9
K_2_O:N = 2	37.87 ± 3.8	34.00 ± 3.6	32.64 ± 3.3 a	29.51 ± 9.9
*P*-value	ns	ns	0.013^*^	ns
*P*-value interaction D × F	ns	ns	ns	ns
**INFRUCTESCENCE LENGTH (cm)**				
**Planting density (D)**				
Low (54,400 plants.ha^−1^)	17.02 ± 2.6	16.47 ± 2.5	15.79 ± 2.1	14.25 ± 1.9
Medium (66,600 plants.ha^−1^)	16.79 ± 2.7	16.15 ± 2.4	15.85 ± 2.2	14.24 ± 2.1
High (74,000 plants.ha^−1^)	16.83 ± 2.7	15.75 ± 2.4	15.29 ± 2.2	13.95 ± 2.0
*P*-value	ns	ns	ns	ns
**Fertilizer (F)**				
K_2_O:N = 0.35	16.88 ± 2.9	16.03 ± 2.5	15.04 ± 2.2 b	13.67 ± 2.0 b
K_2_O:N = 1	17.17 ± 2.5	15.98 ± 2.5	16.41 ± 2.2 a	14.23 ± 2.0 ab
K_2_O:N = 2	16.59 ± 2.6	16.38 ± 2.4	15.48 ± 1.9 b	14.54 ± 1.9 a
*P*-value	ns	ns	0.004^**^	0.023^*^
*P*-value interaction D × F	ns	ns	ns	ns
**CROWN LENGTH (cm)**				
**Planting density (D)**				
Low (54,400 plants.ha^−1^)	21.15 ± 3.0	17.77 ± 2.6	17.12 ± 2.6	15.19 ± 2.3
Medium (66,600 plants.ha^−1^)	21.00 ± 2.5	17.30 ± 2.6	16.73 ± 2.6	14.84 ± 2.8
High (74,000 plants.ha^−1^)	21.21 ± 2.8	17.15 ± 2.6	16.68 ± 2.5	15.15 ± 8.2
*P*-value	ns	ns	ns	ns
**Fertilizer (F)**				
K_2_O:N = 0.35	20.94 ± 2.8	17.28 ± 2.8	16.56 ± 2.6	15.11 ± 8.4
K_2_O:N = 1	21.08 ± 2.5	17.31 ± 2.7	16.82 ± 2.5	15.10 ± 2.5
K_2_O:N = 2	21.33 ± 3.0	17.63 ± 2.3	17.16 ± 2.6	14.97 ± 2.3
*P*-value	ns	ns	ns	ns
*P*-value interaction D × F	ns	ns	ns	ns
**RATIO CROWN: INFRUCTESCENCE LENGTH**				
**Planting density (D)**				
Low (54,400 plants.ha^−1^)	1.28 ± 0.2	1.11 ± 0.2	1.10 ± 0.2	1.08 ± 0.2
Medium (66,600 plants.ha^−1^)	1.29 ± 0.2	1.10 ± 0.2	1.07 ± 0.2	1.07 ± 0.2
High (74,000 plants.ha^−1^)	1.30 ± 0.3	1.11 ± 0.2	1.11 ± 0.2	1.10 ± 0.5
*P*-value	ns	ns	ns	ns
**Fertilizer (F)**				
K_2_O:N = 0.35	1.28 ± 0.3	1.11 ± 0.2	1.12 ± 0.2 a	1.12 ± 0.5
K_2_O:N = 1	1.26 ± 0.2	1.11 ± 0.2	1.04 ± 0.2 b	1.08 ± 0.2
K_2_O:N = 2	1.33 ± 0.3	1.10 ± 0.2	1.13 ± 0.2 a	1.05 ± 0.2
*P*-value	ns	ns	0.018^*^	ns
*P*-value interaction D × F	ns	ns	0.018^*^	ns

*ns, not significant (p ≥ 0.05)*;

* *Significant at 0.01 ≤ p < 0.05*;

** *Significant at 0.001 ≤ p < 0.01*;

*** *Significant at p < 0.001. Means within a column followed by different letters are significantly different*.

A main effect of the K_2_O:N ratio on the fruit weight was observed in three out of the four experiments (Experiments 2–4). The total weight per fruit increased with an increase in the K_2_O:N ratio, except in Experiment 1, where the differences were not significant (Table 7). The infructescence weight also increased with an increase in the K fertilizer application, but only in Experiments 2 and 4. In Experiments 1 and 3, no significant effect of the K_2_O:N ratio on the infructescence weight was observed (Table 7). There were no effects of the K_2_O:N ratio on the crown weight in all experiments (Table 7).

#### Fruit Length, Infructescence Length, Crown Length, and Ratio Crown: Infructescence Length

In all experiments, the planting density did not affect the fruit length, infructescence length, or crown length (Table 7).

A main effect of the K_2_O:N ratio on the fruit length was only significant in Experiment 3. In that experiment, fruits from the plants fertilized with K_2_O:N ratios of 1 and 2 were longer than those from the plants grown with a K_2_O:N ratio of 0.35. For the infructescence length, significant effects were observed in two experiments (Experiments 3 and 4) where plants produced the fruits with the longest infructescences when grown with a K_2_O:N ratio of 1 (Experiment 3); or with a K_2_O:N ratio of 1 or 2 (Experiment 4; Table 7). The K fertilizer application had no significant effect on the crown length.

**Figure 3:**
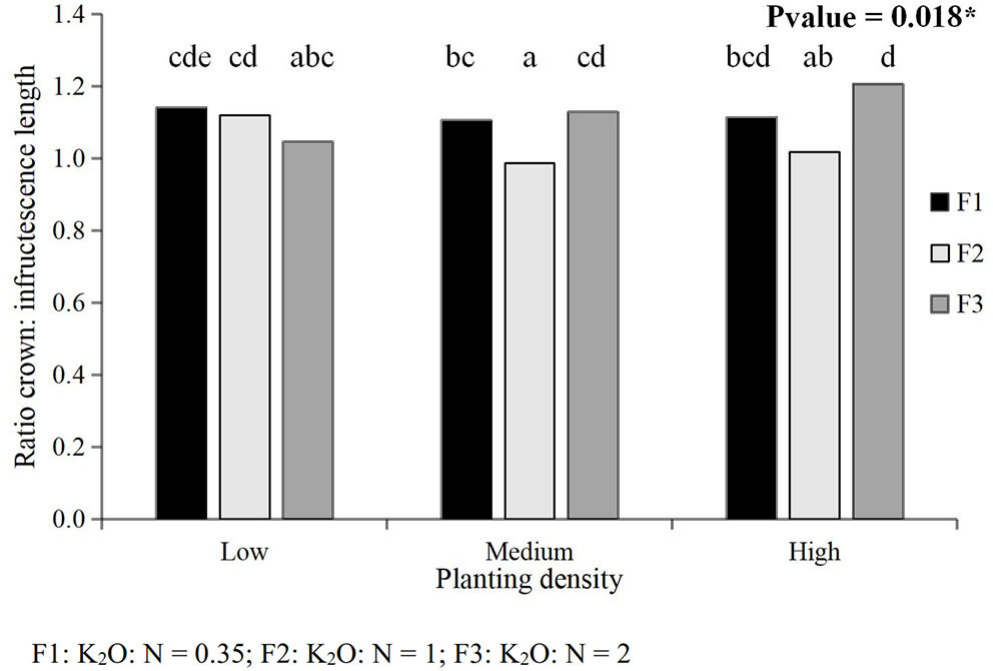
Interacting effects of the planting density and K_2_O:N ratio on the ratio crown: infructescence length in Experiment 3. *Significant at 0.01 ≤ *p* < 0.05. Similar letters at the top of the bars indicate that differences in the means of the treatment combination are not significant.

There were no significant effects of the planting density and fertilizer regime on the ratio crown length: infructescence length except for an interaction between the factors in Experiment 3. Plants grown at high planting density combined with a K_2_O:N ratio of 2 showed the highest crown length: infructescence length ratio while plants grown at a medium planting density with a K_2_O:N ratio of 1 showed the lowest ratio (Figure 3).

#### Effects on Shelf Life

The planting density had no significant effect on the traders' perceived fruit shelf life regardless of the planting season (Table 8). A main effect of K fertilizer on the shelf life of the pineapple fruits was observed regardless of the planting season. Fruits from the plants grown at a K_2_O:N ratio of 1 showed the longest shelf life (Table 8), whereas fruits from the plants grown at a K_2_O:N ratio of 0.35 showed the shortest shelf life (Table 8). The shelf life of fruits from the plants grown at a K_2_O:N ratio of 2 as perceived by traders was statistically similar to that of the fruits grown with a K_2_O:N ratio of 1 (Table 8).

**Table 8:** Effects of planting density and K_2_O:N ratio on the pineapple fresh fruit shelf life (days) in experiments planted in different seasons; data are presented as average ± SD.

**Source of variation**	**Long rainy season (Exp. 1)**	**Short rainy season (Exp. 3)**
**Planting density (D)**		
Low (54,400 plants.ha^−1^)	7.89 ± 3.06	7.33 ± 2.91
Medium (66,600 plants.ha^−1^)	7.89 ± 3.55	8.67 ± 3.20
High (74,000 plants.ha^−1^)	7.67 ± 2.78	8.22 ± 2.68
*P*-value	ns	ns
**Fertilizer (F)**		
K_2_O:N = 0.35	4.44 ± 0.88b	4.66 ± 1.00b
K_2_O:N = 1	10.11 ± 1.83a	10.00 ± 1.73a
K_2_O:N = 2	8.89 ± 2.34a	9.55 ± 1.81a
*P*-value	0.033^*^	0.014^*^
*P*-value interaction D × F	ns	ns

*ns, not significant (p ≥ 0.05)*;

* *Significant at 0.01 ≤ p < 0.05. Means within a column followed by different letters are significantly different*.

## Discussion

The objective of our study was to quantify the effects and interaction of agronomic practices, i.e., planting density and K_2_O:N ratio, on the pineapple yield and quality attributes including the shelf life. To better explain how these agronomic practices affect these variables, it was important to depict how they affect the crop developmental variables at the moment of flowering induction. Such line of reasoning was supported by the results of the studies of Fassinou Hotegni et al. (2014), who demonstrated that the heterogeneity of the pineapple fruit quality (external fruit quality, mainly fruit weight) is a consequence of the heterogeneity in the vigor of the plants at the moment of flowering induction.

### Effect of the Planting Density and Fertilizer Management on Crop Developmental Variables

The results indicated that the number of functional leaves and the D-leaf length at the moment of flowering induction were affected neither by the planting density nor by the K_2_O:N ratio. The same observations were made for the leaf area of the D-leaf and the weight of the D-leaf except in Experiment 1 (Table 5). Similar observations on some variables, such as the number of functional leaves and the D-leaf length were made by some authors. Dass et al. (1978), who worked with cv. Kew with the density ranging from 49,382 to 111,111 plants.ha^−1^, reported no effect of the planting density on the number of functional leaves just before the flowering induction. Maia et al. (2009) reported no effect of the planting density (ranging from 41,666 to 55,555 plants.ha^−1^) on the D-leaf length of the “Perola” pineapple cultivar just before the flowering induction. Hung et al. (2011) also reported the same results for the D-leaf length of Smooth Cayenne using the planting density ranging from 57,000 to 78,000 plants.ha^−1^. The observed lack of effect of the planting density and K_2_O:N ratio on the number of functional leaves and D-leaf morphology suggests that the leaf development rate was not affected. This implies that the planting density used in our experimentation might not be high enough to induce competition among plants for mainly light and water in the soil, leading to a neutral interaction among plants. In our study, only the K_2_O:N ratios of 0.35 and 1 were applied before the flowering induction. Since a difference between these two treatments is based on additional application of K applied 3 months before the flowering induction (Table 2), a slight increase in the D-leaf length and/or area could be expected but the observed changes did not lead to a significant difference in the measured crop development variables except in Experiment 1 for the D-leaf area only (Table 5). It can be inferred that the additional amount of K applied was not sufficient enough to induce visible changes in the crop developmental variables at the moment of flowering induction.

### Effect of Planting Density and K_2_O:N Ratio on Pineapple Fruit Yield

In all experiments, increasing the planting density increased the total yield (Table 6). Such results are in line with those reported by Norman (1978) and Mukherjee et al. (1982). However, in the studies reported by these authors, the increase in the yield was at the expense of the fruit weight. In our study, no effects of the planting density on the total weight per fruit and infructescence weight were observed (Table 7). An increase in the pineapple fruit yield was a consequence of an increase in the plant number per meter squared with the highest fruit yield obtained in the pineapple fields established in the long rainy season. Moreover, the pineapple fruit yields obtained from the plants grown at 66,600 plants.ha^−1^ were not significantly different to those obtained from the plants grown at 74,000 plants.ha^−1^ despite an increase in the number of plants by 11%. From this observation, it can be inferred that 66,600 plants.ha^−1^ could be the optimum pineapple planting density when considering the yield since adding more plants would increase production cost without any significant increase in the yield.

In all experiments, the K_2_O:N ratio affected the total yield except in Experiment 1 (Table 6) with an increase in the yield in line with an increase in the K_2_O:N ratio. This was expected since the K_2_O:N ratio was found to improve the total weight per fruit in three out of four experiments (Table 7).

### Effect of the Planting Density and K_2_O:N Ratio on External Fruit Quality at Harvest and Shelf Life

#### Fruit Quality at Harvest

There were no effects of the planting density on the total fruit weight, infructescence weight, total fruit length, infructescence length, and crown length in all experiments except in Experiment 2 where the crown weight was the highest at the lowest plant density (Table 7). This lack of effects of the planting density on quality attributes was not expected since it is well-known that, at high planting density, plants would compete for light and water in the soil, reducing the capacity of plants grown at high density to produce high-quality fruits. Similar results were reported in the literature. Maia et al. (2009), with the planting density ranging from 41,666 to 55,555 plants.ha^−1^ for cv. Pérola (a cultivar close to cv. Sugarloaf), reported no effect of the planting density on the fruit weight, infructescence weight, and fruit length. Hung et al. (2011) also reported no effect of the planting density on the average cv. Smooth Cayenne fruit weight when the planting density ranged from 57,000 to 66,000 plants.ha^−1^. Above the value of 66,000 plants.ha^−1^, a decrease in the average total fruit weight was observed. Mohammed Selamat (1995), who worked on cv. Gandul with the planting density varying from 43,056 to 61,508 plants.ha^−1^, reported no effect on the average fruit weight and fruit length. Although the research by Maia et al. (2009) reported no effect of increasing the planting density on the fruit length, there was no evidence in the literature on how the fruit length components could be affected by increasing the planting density. Our results provide evidence that the lack of effect of the planting density (in the range of our study) on the total fruit length is a consequence of the lack of effect on the infructescence length and crown length (Table 7). The observed and consistent lack of effect of the planting density on the weight and length attributes are in line with the lack of effect of increasing the planting density on the plant development variables at the flowering induction time. Fassinou Hotegni et al. (2015a) have demonstrated that within a pineapple field, there is a clear association between the vigor of the plant at the flowering induction time and the fruit quality at harvesting.

In three out of the four experiments, the K_2_O:N ratio had an effect on the total fruit weight with increasing K_2_O:N ratio, leading to a significant increase in the fruit weight. Such an increase in the fruit weight was mainly a consequence of an increase in the infructescence weight (a significant increase in the infructescence weight observed in two experiments and a non-significant increase observed in the other two experiments) since no effect of the K_2_O:N ratio on the crown weight was observed (Table 7). Such effects of K fertilization on the fruit weight and infructescence weight can be explained by a difference in the additional and cumulative effect of K applied just before and after the flowering induction. It is well-known that K participates in crop photosynthesis by not only facilitating the nutrients and water uptake and transport through the xylem but also most importantly facilitating the translocation of photosynthates from leaves to sinks, such as growing fruits (Zörb et al., 2014). So, in the treatments where the K_2_O:N ratio is high, K availability in the plant tissue would help translocate photosynthates to a fruit, which would lead to an increase in the fruit size. Swete Kelly (1993) pointed out that the deficiency in K in the pineapple cultivation leads to a weak peduncle, then reducing the capacity of the peduncle to maintain big fruits. Therefore, it can be inferred that the fruit weight in pineapple is mostly determined by the capacity of the plant to direct photosynthates to growing sinks (crop nutrition with K) than by a strong vegetative development at the flowering induction time. Regarding the fruit length, in three out of the four experiments, no significant effect of the K_2_O:N ratio was observed (Table 7). This may be explained by the fact that, with K fertilization, the fruit tends to increase in width rather than in length. The observation that the crown weight and its length did not change significantly in response to the K_2_O:N ratio can be explained by the fact that the crown as vegetative organs may have either a poor sink strength compared to the infructescence or the crown, as demonstrated by Fassinou Hotegni et al. (2015b) with slips, or may be autotrophic in terms of photosynthates for its growth.

In three out of the four experiments, the crown: infructescence length ratio was not affected by the two factors. This is a consequence of the lack of “consistent” effect of the two factors on the crown length and infructescence length.

#### Shelf Life

Shelf life is also a very important quality attribute. Of the two factors, and regardless of the planting season, only the K_2_O:N ratio had an effect on the fresh pineapple shelf life with plants receiving a K_2_O:N ratio of 1 giving fruits showing a 6-day longer shelf life than plants from the farmers' fertilization practices (ratio of 0.35; Table 8). An increase in the K_2_O:N ratio to 2 did not significantly affect the shelf life of the fruits compared to that observed with a K_2_O:N ratio of 1. An increase in the shelf life of pineapple fruits as a consequence of an increase in the K_2_O:N ratio from 0.35 to 1 can be explained by the ability of K to increase the sink strength (i.e., the capacity of the fruit to take available assimilates), and fruit firmness, a proxy of the fruit shelf life (Mikkelsen, 2018). Such an effect of K fertilization has also been reported in other horticultural crops, such as tomatoes (*S. lycopersicum*) (Passam et al., 2007) and pepper (*C. annuum*) (Botella et al., 2017) where an increase in K improved the firmness, ascorbic acid concentration, TSS, and soluble sugars. However, further studies should focus on matching the traders' criteria with internal quality traits of the fruits, such as changes in ethanol, TSS, and total titratable acid having an objective definition of the end of the shelf life.

## Conclusions

The effects of agronomic practices, such as planting density and K_2_O:N ratio, on the fruit quality are important in the pineapple cultivation. Our experiments showed that the planting density in the range of our study (54,400, 66,600, and 74,000 plants.ha^−1^) and the K_2_O:N ratio had no effect on the crop developmental variables just before the flowering induction. Fruit quality attributes, such as fruit weight, infructescence weight, total fruit length, infructescence length, and crown length were not significantly affected by the planting density. On the other hand, the fruit weight was positively affected by an increase in the K_2_O:N ratio. The planting density had no effect on the fruit shelf life. An increase in the K_2_O:N ratio from 0.35 to 1 improved the shelf life of the fruits by almost 6 days. An increase in the planting density up to 66,600 led to a significant increase in the fruit yield; further increase was not significant. The K_2_O:N ratio also improved the fruit yield through an improvement of the fruit weight.

## Data Availability Statement

The dataset generated during and/or analyzed in the present study is available from the authors upon request.

## Author Contributions

UD, NVFH, WJML, and PCS conceived and designed the experiments. UD, NVFH, and EGA-D performed the experiments. UD, NVFH, WJML, EGA-D, and PCS analyzed the data. UD, NVFH, WJML, EGA-D, JDH, and PCS wrote the manuscript. UD, NVFH, WJML, EGA-D, JDH, and PCS performed critical revisions. All authors read and approved the final manuscript.

## Conflict of Interest

The authors declare that the research was conducted in the absence of any commercial or financial relationships that could be construed as a potential conflict of interest.

## Abbreviations

TSS: total soluble solids.
